# scFv against HSP60 of *Strongyloides* sp. and Its Application in the Evaluation of Parasite Frequency in the Elderly

**DOI:** 10.1155/2020/4086929

**Published:** 2020-01-11

**Authors:** Camila Botelho Miguel, Marcelo Arantes Levenhagen, Julia Maria Costa-Cruz, Luiz Ricardo Goulart, Patrícia Terra Alves, Carlos Ueira-Vieira, Patrícia Kellen Martins Oliveira Brito, Angelica Oliveira Gomes, Javier Emilio Lazo-Chica, Carlo José Freire Oliveira, Wellington Francisco Rodrigues

**Affiliations:** ^1^Federal University of Triângulo Mineiro (UFTM), 38061-500 Uberaba, MG, Brazil; ^2^University Center of Mineiros-Unifimes, 75., 830-000 Mineiros, GO, Brazil; ^3^Federal University of Uberlândia, 38400-902, Uberlandia, MG, Brazil; ^4^University of São Paulo, 14049900, Ribeirao Preto, SP, Brazil

## Abstract

The present study is aimed at evaluating serological method using scFv anti-*Strongyloides* sp. and reporting the frequencies of the results with conventional parasitological technique (faeces) in elderly individuals. Among 112 elderly individuals (≥60 years of age), 14.28% were positive for at least one enteroparasite, with one individual positive for *S. stercoralis*. Sera were evaluated for the presence of anti-*Strongyloides* sp. antibodies using total or detergent fraction extracts of *Strongyloides venezuelensis*, which presented positivity rates of 19.64% and 10.71%, respectively. An anti-HSP60 single-chain variable fragment from *Strongyloides* sp. was used to detect parasite antigens, with 5.36% (6 individuals) of ELISA-positive individuals returning a positive result. While the serological test indicates previous or recent infection and may be limited by antigen purification, the anti-HSP60 method reflects the presence of *Strongyloides* sp. immune complexes and exhibits greater sensitivity and specificity. Our results demonstrate the variable occurrence of enteroparasites in elderly individuals residing in long-term nursing homes and validate a novel epidemiological tool to describe infection cases by *Strongyloides* sp.

## 1. Introduction

Among the pathogenic helminths investigated, the one most often diagnosed is *Strongyloides stercoralis* (*S. stercoralis*), a nematode parasite that causes strongyloidiasis, a disease characterized by skin and digestive symptoms, in humans [1–5]. Parasitological surveys using more sensitive and rigorous techniques are needed to provide more reliable results and serve as the basis for future interventions involving sanitary and educational measures to improve health maintenance [2]. In this sense, research carried out for the refinement of techniques applied to the epidemiological survey to parasitic diseases, such as the strongyloidiasis, is necessary. It should be noted that infection by this parasite is becoming increasingly severe in vulnerable groups, such as immunosuppressed patients, children, and the elderly [5–7]. The major concern in these vulnerable groups is the low immune capacity to respond effectively to infectious processes. In the case of the elderly, this problem is increasing as this population experiences considerable growth and longer life expectancy; these changes will have profound impacts on public health in the coming decades.

One of the factors that is associated with quality of life and an increase in the number of elderly people worldwide is the high demand for long-term institutions known worldwide as retirement homes. In these environments, physical and social structures have particular characteristics that may be associated with the emergence or control of infectious and/or parasitic diseases, including by *S. stercoralis*.

Studies to diagnose the incidence and/or prevalence of parasitic diseases at these institutions have been conducted over the years. For the diagnostic tests available and implemented so far, the results show that the prevalence of enteroparasites in the elderly is not associated with long-term institutions, sociodemographic characteristics, lifestyle, or health conditions [4, 5]. However, the frequencies of enteroparasites in the elderly appear to be underestimated because they may vary depending on the study and the country/region evaluated.

The choice and availability of techniques with greater specificity and good sensitivity for the screening of *S. stercoralis* are limiting factors for the precise diagnosis and epidemiological analysis of the disease, leading to underestimates of *S. stercoralis* infections [1, 8]. From among the existing techniques, the Baermann technique, involving the use of agar plate culture, contributed to an increase in the specificity of the detection of *Strongyloides* in the faeces, but it still exhibits a variable sensitivity due to the scarcity of larvae in many infections and the amount of faecal material collected and evaluated [1, 9]. Serological techniques represent promising alternatives in the search for greater diagnostic sensitivity. However, these techniques still present some limitations, such as crossreactions that lead to the antigenic recognition of other nematodes and compromise the diagnosis of these endoparasites [1, 9]. Therefore, there is an ongoing search for more efficient and safer methods of detection.

Recently, a study from our research group presented a serological method for the detection of immunocomplexes formed from the binding of a single-chain variable fragment (scFv) to a specific protein from *Strongyloides* sp., HSP60. This serological method of diagnosis demonstrated a sensitivity of 97.5% and specificity of 98.81% to *Strongyloides* sp. [10]. The anti-*Strongyloides* scFv was incorporated into this test after the use of phage display, a fast and reliable technique that allows for the selection of peptides, antibodies, or scFvs highly specific for a particular pathogen. Thus, the characteristics of this method enabled the discovery of a molecule with important diagnostic applicability due to its high specificity and ease of production [11]. In this study, we aimed to demonstrate the use of the newly developed technique for the detection of immunocomplexes of Strongyloides sp. We also used this serological and conventional method to evaluate the frequency of enteroparasites in elderly individuals living in long-term residences.

## 2. Material and Methods

### 2.1. Ethics

All procedures related to this research were approved by the research ethics committee of the Federal University of Triângulo Mineiro (number: 017430/2014) and are registered in Plataforma Brasil in accordance with resolution 466/2012 of the National Health Council.

### 2.2. Inclusion and Exclusion Criteria

For this study, 112 individuals of both sexes who were ≥60 years of age and who resided in long-term residences in the city of Uberaba, Minas Gerais, Brazil, were enrolled. Patients with unsatisfactory samples (failure to obtain at least three faecal samples and/or to obtain a serum sample) were excluded from the evaluation.

### 2.3. Biological Samples

Three faecal samples were collected on alternate days for a period of 7 days. Collection was carried out in labelled sterile plastic collectors, and a small portion (5 g) was used for larval research while the rest was stored in flasks containing 10% buffered formaldehyde. In addition, the peripheral blood was collected (dry tube) to obtain serum by centrifugation at 1831 × *g* for 10 min. Sera were frozen at –80°C until use.

### 2.4. Detection of Enteroparasites in Faeces

Two methods were used to detect enteroparasites in the faeces: a spontaneous sedimentation test (Hoffman test) [12] and the Baermann-Moraes test [13]. The Hoffman method was used to detect larvae, helminth eggs, and protozoan cysts. For each individual, about 5 g of faeces was dissolved in 10 mL of water in a small vial, and then, the sample was filtered through four-part folded gauze using a sedimentation cup. These samples were incubated for 24 h. With the help of a pipette, the sample was removed from the apex of the chalice for evaluation. The material was stained with Lugol's solution and examined under a light microscope (40x). For the Baermann-Moraes method, water at 40°C was added to a glass funnel until the level reached 1/2 the height, at which point it was connected to a rubber tube and closed with forceps, so the sample was contained. Then, the gauze was placed with the faeces on a strainer in contact with the funnel and water, so that the faeces were submerged for a few minutes at rest. Later, the forceps were removed to collect the liquid. After transferring to a slide, the presence of larvae was evaluated under a light microscope (40x).

### 2.5. Detection of Anti-*Strongyloides* Antibodies

Anti-*Strongyloides* sp. antibodies in all samples were detected using a total or partial (fraction) extract of *Strongyloides venezuelensis* (fusiform larva, stage 3). The production of the total and partial extracts was performed according to the methods of Da Silva et al. [14], and immunoenzymatic assays were performed as described below. For the detection of antibodies, high-affinity polystyrene plates (BioAgency Laboratories, São Paulo, Brazil) were coated with 5 *μ*g/mL of total salt extract (ES) or the detergent fraction (D) and incubated overnight at 4°C in 0.06 M bicarbonate buffer, pH 9.6. After incubation, the plates were washed three times for 5 min each with phosphate-buffered saline (PBS) plus 0.05% Tween 20 (PBS-T) and blocked with PBS-T plus 3% skim milk at 37°C for 45 min. Serum samples (diluted 1 : 80) were added and incubated for an additional 45 min at 37°C. After washing with PBS-T, peroxidase-conjugated anti-human IgG antibody (1 : 2000) was added and incubated for 45 min at 37°C. The reaction was revealed by the addition of enzyme substrate (0.03% H_2_O_2_) and chromogen (*o*-phenylenediamine (OPD)) in 0.1 M phosphate-citrate buffer (pH 5.0). The reaction was incubated for 15 min at 24°C and stopped by the addition of 2 N NH_2_SO_4_. Previously known positive and negative samples were used as controls of the analytical run. Optical densities were determined at 492 nm in an enzyme-linked immunosorbent assay (ELISA) reader (Titertek Plus, Flow Laboratories, McLean, VA, USA).

### 2.6. Detection of Immunocomplexes in Human Sera Using scFv against *Strongyloides* sp. HSP60

For the detection of *Strongyloides* sp. immunocomplex in the sera of the elderly individuals included in the study, an enzymatic immunoabsorption assay was performed. To this end, high-affinity microtiter plates (Nunc MaxiSorp™, Thermo Fisher Scientific, Waltham, MA, USA) were incubated with 50 *μ*L anti-HSP60 scFv (10 *μ*g/mL) in bicarbonate buffer (0.06 M, pH 9.6) overnight at 4°C. Plates were blocked with 5% PBS/bovine serum albumin (BSA) for 45 min at 37°C. Serum samples were diluted 1 : 50 in PBS-T and added to the wells, and the plates were incubated for 45 min at 37°C. Subsequently, peroxidase-conjugated human anti-human IgG diluted in PBS-T (1 : 10,000 dilution) was added to the wells, and the plates were incubated for 45 min at 37°C. Between the steps, three washes were performed with PBS-T. The reaction was revealed by the addition of OPD diluted in 0.1 M citrate-phosphate (pH 5.0) and 30% H_2_O_2_. Plates were incubated for 15 min at room temperature, and the reactions were quenched by the addition of 2 N H_2_SO_4_. Optical densities (OD) were determined at 492 nm on an ELISA reader (Titertek Plus, Flow Laboratories). Under these conditions, the TG-ROC curve obtained the best cut-off (0.7175), as already described in another study [10].

### 2.7. Research Quality Control

Internal quality control processes were incorporated into the study where there was a clear definition of objectives, procedures, criteria for tolerance limits, corrective actions, and recording of activities. Controls to evaluate the imprecision of the analyses were also monitored at the preanalytical, analytical, and postanalytical phases [15, 16].

### 2.8. Statistical Analysis

Statistical analysis was performed using Prism software (GraphPad Inc., San Diego, CA, USA) and Excel (Microsoft). The chi-square test was used to determine the statistical significance of the data, with differences with *p* < 0.05 (5%) considered significant.

## 3. Results

### 3.1. Detection of Enteroparasites in Faeces Using the Hoffman and Baermann-Moraes Methods

A total of 112 individuals with a mean age of 76 years (range 60–109) were analysed. For each individual, three faecal samples were obtained on alternate days over a period of 7 days. Peripheral blood samples were also collected to obtain serum (between faecal collection days). First, the presence of enteroparasites in the faeces of the elderly individuals was determined. Only 14.28% of the individuals (16 individuals) tested positive for at least one parasite in the faeces. Among the positive individuals, 81.25% (13 individuals) were positive for only one type of enteroparasite, and the other 18.75% (3 individuals) were positive for at least two species. The prevalence of enteroparasite species among the faecal samples of 16 positive individuals were as follows: *Entamoeba coli* (10 individuals), *Giardia lamblia* (2 individuals), *Endolimax nana* (2 individuals), *Blastocystis hominis* (1 individual), and *S. stercoralis* (1 individual). The frequencies of enteroparasite occurrence were also evaluated separately in the sexes, but no significant differences were observed in the samples evaluated.

Detection of anti-*Strongyloides* sp. IgG antibodies using total and partial extracts of *S. venezuelensis.*

**Figure 1:**
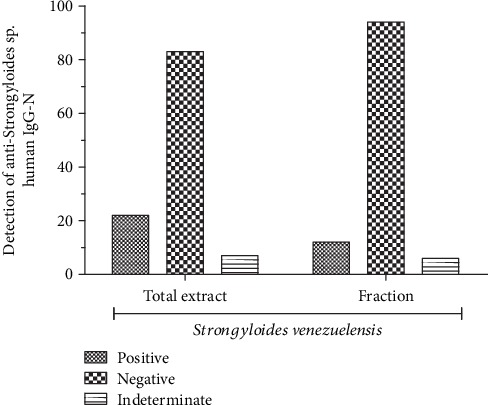
Positivity rates for anti-*Strongyloides* sp. antibodies among individuals residing in long-term institutions. After obtaining whole blood from elderly individuals living in long-term residences, samples were centrifuged to obtain the serum, and ELISA was performed. Plate sensitization was performed with the use of a total and/or partial extract of *Strongyloides venezuelensis*. Frequencies (positive, negative, and undetermined) were compared between the total and partial extract. No significant differences, chi-square test (*p* > 0.05).

In the above experiment, we detected *S. stercoralis*, an important enteroparasite related to morbidity and mortality in the elderly. However, because the parasitological test does not exhibit high sensitivity, we additionally tested whether we could detect the presence of anti-*Strongyloides* sp. IgG antibodies in the sera of the 112 individuals in this study. For detection, we tested the sera of the 112 patients against a total or partial extract of the parasite *S. venezuelensis*. Among the 112 individuals, 19.64% (22 individuals) were positive for anti-*Strongyloides* sp. IgG antibodies and 6.25% (7 individuals) of the samples were indeterminate using the total *S. venezuelensis* extract. When we used only the partial extract, 10.71% (12 individuals) of the samples were positive and 4.46% (5 individuals) were indeterminate. Importantly, although the use of the partial extract resulted in greater specificity, it was not significantly different from that of the total extract (Figure 1).

### 3.2. Detection of Immunocomplexes in Individuals Positive for Anti-*Strongyloides* sp. IgG Antibodies

The presence of IgG antibodies against *Strongyloides* sp. HSP60, per se, does not necessarily indicate clinical symptoms in the elderly, since an individual may have had contact with the parasite at any time over their lifetime. Recently, our group developed a serological method for the detection of immunocomplexes formed from the binding of an scFv to the *Strongyloides* sp. HSP60 protein. Therefore, we sought to describe epidemiological data, after specific immune response already established against *Strongyloides* sp. in the elderly. We evaluated the presence of immunocomplexes in the sera of the 22 individuals that were positive for IgG antibodies against *Strongyloides* sp. HSP60. Interestingly, 27.27% of these 22 elderly individuals tested exhibited the presence of immunocomplexes (*n* = 6). This represents 5.36% of the total population of 112 individuals enrolled in the study. The frequency of each result (positive, negative, and indeterminate) was compared among the serological tests (total extract, partial extract, and immunocomplex) and parasitological tests, which can be observed in Table 1. The detection of immunocomplexes for *Strongyloides* sp. by serological testing exhibited greater sensitivity and specificity.

## 4. Discussion

Enteroparasitoses are neglected diseases that affect millions of people worldwide, including the elderly, a population that has increased in number in recent decades [17]. Surveys of medically important enteroparasites show high rates of *S. stercoralis* infection among the populations that have been evaluated [4, 5, 18]. Among the various populations that have been studied, important findings have been observed in the elderly, especially those institutionalized in long-term residences [4, 18]. Despite these results, the World Health Organization has expressed concern over data involving the diagnosis of strongyloidiasis, and this may be due to the absence and/or imprecision of diagnostic tools [1]. In this study, we evaluated the most commonly used diagnostic methods, besides evaluating the application of a serological test for the detection of immunocomplexes previously developed by our research group. Our findings suggest that conventional tests (parasitological evaluation of faeces) may generate inaccurate and inferior data. However, the only test that would determine the incidence of *Strongyloides sp*. infection in the elderly in this study was the Hoffman test and the Baermann-Moraes test, which although they gave a low incidence, we cannot conclude that their sensitivity and specificity are less than the ELISA or the immune complex test, because they measure periods within the different parasite cycle stage.

Our results also enabled the evaluation of the frequency of enteroparasites in elderly individuals living in long-term residences, demonstrating that the newly developed test has an optimal level of safety independent of the evaluated population.

Our data showed that of the 112 individuals tested, the positivity rates for the conventional tests were very low for most of the endoparasites tested, including *S. stercoralis*. In contrast, the rate of detection of anti-*Strongyloides* sp. antibodies using the ELISA-based method and sensitization of the ELISA plates with total *S. venezuelensis* extract was higher; although the test is not able to demonstrate the early onset of infection, it is able to indicate the humoral adaptive immune response installed in previously infected individuals. The higher positivity rate associated with total rather than partial *S. venezuelensis* extract may reflect crossreactions to other antigens, which has been previously discussed in other studies [1, 10, 19–21].

Importantly, 27.27% (*n* = 6) of individuals positive for anti-*S. stercoralis* IgG antibodies were also positive for the presence of immunocomplexes. If we compare this with the use of the conventional faecal test, only one individual was positive for *S. stercoralis*, and this same individual was among those positive for the presence of immunocomplex. Thus, we believe that these techniques complement each other, as the parasite in question has a biological cycle that includes diverse forms due to its developmental and larval stages and may be linked to haematological and allergic manifestation [22].

Frequent efforts of the scientific community have been made to improve the diagnostic accuracy of tests for different enteroparasites of medical interest, including *S. stercoralis* [10, 23, 24]. To understand the need for these new diagnostic or epidemiological study tools, a recent comparative study with 98 samples concluded that there were discrepancies between molecular tests (real-time PCR) and microscopic methods for the diagnosis of 20 enteroparasites [25]. As previously mentioned, the method for the detection of immunocomplexes using the anti-*Strongyloides* sp. scFv ELISA assay showed excellent specificity (98.81%) and sensitivity (97.5%) [10], which is highly relevant if we take into account the diagnostic capacity of this tool, after humoral adaptive immune response, specifically for this disease.

This test in addition to its diagnostic contributions serves studies like this one provide epidemiological data that contribute to the improvement of health services, enabling the selection of appropriate forms of intervention for the control of these parasitoses [2].

Recent evaluations contribute to demonstrate the need to increase diagnostic sensitivity in the strongyloidiasis. One of the alternatives evaluated was the use of molecular biology techniques such as the real-time polymerase chain reaction (PCR), where it was shown to be more sensitive than the conventional parasitological test [26]; on the other hand, a sensitivity of 63% of qPCR for the diagnosis of *S. stercoralis* in faeces and 17% in urine has been demonstrated, indicating that the sensitivity is varied and there is a need for more implementations for the validation of the technique [27]. We thus believe that the detection of serum immunocomplexes can contribute to the diagnosis and for epidemiological surveys, quickly and inexpensively, in association with other techniques, including the detection of parasites in faeces.

The results of this survey indicated a 14% positivity rate among the evaluated individuals for at least one endoparasite, including rates of *Entamoeba coli*, *G. lamblia*, *Endolimax nana*, *B. hominis*, and *S. stercoralis* of 65%, 15%, 10%, 5%, and 5%, respectively. These results differ somewhat from previously published results. For example, in a study of 183 subjects conducted in the southwestern region of Saudi Arabia, the rate of positivity for enteroparasites was 70.5% and the highest prevalence was found in individuals under the age of 30 [28]. In this study, the researchers observed a high frequency of amoebas associated with amebiasis (*Entamoeba histolytica* and *Entamoeba dispar*). In contrast, in our survey, the highest incidence was for the commensal protozoan *Entamoeba coli*. Commensal bacteria and protozoa are commonly found to be associated with enteroparasites in epidemiological studies [2]. Another study reported frequencies of *Entamoeba coli* of 47.5% among individuals in long-term institutions and 60.9% among geriatric outpatients [2]. The same researchers reported a frequency of enteroparasites of 12.9% among institutionalized elderly. Thus, although frequencies appear to vary, parasitic infections along with bacterial infections remain a serious public health problem.

Data on the distribution of intestinal parasitic infections in the metropolitan area of Rio de Janeiro, Brazil, also indicate a significant number of enteroparasitoses, including in the elderly [18]. In one study, the authors found an association between the socioeconomic status of the population and the incidence of infections, demonstrating that the infection rate may track socially vulnerable areas [18]. In the same study, the authors observed a high rate of *S. stercoralis* among the described helminths. Similarly, our evaluation pointed to *S. stercoralis* as an important helminth, corroborating the data from Naves and Costa-Cruz [4] and emphasizing the need to increase the diagnostic capacity and new epidemiologic reports of these enteroparasites.

## 5. Conclusions

In conclusion, this study provided an analysis of the occurrence and variability of enteroparasites among individuals residing in long-term residences in a city in the south-eastern region of Brazil. In addition, the efficiency of an innovative technique determines the frequency of *Strongyloides* sp. in the elderly and the importance of implementing multiple different techniques in order to increase the sensitivity and specificity of diagnosis. Thus, our results will contribute to the appropriate clinical management and maintenance of elderly health.

## Figures and Tables

**Table 1:** Serological and parasitological analysis of *Strongyloides* sp. in 112 individuals residing in long-term residences.

	Serological			Parasitological
	Total extract	Fraction	Immunocomplex	Faeces
Positive (*n*)	22	12	6	1
Negative (*n*)	83	95	106	111
Indeterminate (*n*)	7	5	0	0
Positivity (%)	19.64	10.71	5.36	0.89

## Data Availability

The data used to support the findings of this study are included within the article.

## References

[1] Bisoffi Z., Buonfrate D., Montresor A. (2013). Strongyloides stercoralis: a plea for action. *PLoS Neglected Tropical Diseases*.

[2] Ely L. S., Engroff P., Lopes G. T., Werlang M., Gomes I., De Carli G. A. (2011). Prevalência de enteroparasitos em idosos. *Revista Brasileira de Geriatria e Gerontologia*.

[3] Gétaz L., Castro R., Zamora P. (2019). Epidemiology of Strongyloides stercoralis infection in Bolivian patients at high risk of complications. *PLoS Neglected Tropical Diseases*.

[4] Naves M. M., Costa-Cruz J. M. (2013). High prevalence of Strongyloides stercoralis infection among the elderly in Brazil. *Revista do Instituto de Medicina Tropical de São Paulo*.

[5] Santos P. H. S., Barros R. C. S., Gomes K. V. G., Nery A. A., Casotti C. A. (2017). Prevalence of intestinal parasitosis and associated factors among the elderly. *Revista Brasileira de Geriatria e Gerontologia*.

[6] Berahmat R., Spotin A., Ahmadpour E. (2017). Human cryptosporidiosis in Iran: a systematic review and meta-analysis. *Parasitology Research*.

[7] Nkenfou C. N., Tchameni S. M., Nkenfou C. N. (2017). Intestinal parasitic infections in human immunodeficiency virus-infected and noninfected persons in a high human immunodeficiency virus prevalence region of Cameroon. *The American Journal of Tropical Medicine and Hygiene*.

[8] Machicado J. D., Marcos L. A., Tello R., Canales M., Terashima A., Gotuzzo E. (2012). Diagnosis of soil-transmitted helminthiasis in an Amazonic community of Peru using multiple diagnostic techniques. *Transactions of the Royal Society of Tropical Medicine and Hygiene*.

[9] Sudré A. P., Macedo H. W., Peralta R. H. S., Peralta J. M. (2006). Diagnóstico da estrongiloidíase humana: importância e técnicas. *Revista de Patologia Tropical*.

[10] Levenhagen M. A., Santos F. A., Fujimura P. T., Carneiro A. P., Costa-Cruz J. M., Goulart L. R. (2015). Erratum: Corrigendum: Structural and functional characterization of a novel scFv anti-HSP60 of _Strongyloides_ sp.. *Science Reports*.

[11] Barbas C. F. (1993). Recent advances in phage display. *Current Opinion in Biotechnology*.

[12] Hoffman W. A., Pons J. A., Janer S. L. (1934). The concentration methods in Schistosomiasis mansoni. *Journal of Public Health*.

[13] Baermann G., Baermann G. (1917). Eine einfache methode zur auffindung von Ankylostomun-(Nematoden)-larven In erdproben. *Mededelingen uit het Geneeskundig Laboratorium te Weltevreden*.

[14] da Silva H., de Carvalho C. J., Levenhagen M. A., Costa-Cruz J. M. (2014). The detergent fraction is effective in the detection of IgG anti-Strongyloides stercoralis in serum samples from immunocompromised individuals. *Parasitology International*.

[15] Henry R. J., Segalove M. (1952). The running of standards in clinical chemistry and the use of the control chart. *Journal of Clinical Pathology*.

[16] Rodrigues W. F., Miguel C. B., Napimoga M. H., Oliveira C. J., Lazo-Chica J. E. (2014). Establishing standards for studying renal function in mice through measurements of body size-adjusted creatinine and urea levels. *BioMed Research International*.

[17] Dall T. M., Gallo P. D., Chakrabarti R., West T., Semilla A. P., Storm M. V. (2013). An Aging Population And Growing Disease Burden Will Require ALarge And Specialized Health Care Workforce By 2025. *Health Affairs*.

[18] Faria C. P., Zanini G. M., Dias G. S. (2017). Geospatial distribution of intestinal parasitic infections in Rio de Janeiro (Brazil) and its association with social determinants. *PLoS Neglected Tropical Diseases*.

[19] Bisoffi Z., Buonfrate D., Sequi M. (2014). Diagnostic accuracy of five serologic tests for Strongyloides stercoralis infection. *PLoS Neglected Tropical Diseases*.

[20] Repetto S. A., Ruybal P., Solana M. E. (2016). Comparison between PCR and larvae visualization methods for diagnosis of _Strongyloides stercoralis_ out of endemic area: A proposed algorithm. *Acta Tropica*.

[21] Requena-Méndez A., Chiodini P., Bisoffi Z., Buonfrate D., Gotuzzo E., Muñoz J. (2013). The laboratory diagnosis and follow up of strongyloidiasis: a systematic review. *PLoS Neglected Tropical Diseases*.

[22] Magnaval J. F., Laurent G., Gaudré N., Fillaux J., Berry A. (2017). A diagnostic protocol designed for determining allergic causes in patients with blood eosinophilia. *Military Medical Research*.

[23] Espírito-Santo M. C., Alvarado-Mora M. V., Pinto P. L. (2015). Comparative study of the accuracy of different techniques for the laboratory diagnosis of Schistosomiasis mansoni in areas of low endemicity in Barra Mansa city, Rio de Janeiro state, Brazil. *BioMed Research International*.

[24] Yanet F. S., Fidel Angel N. F., Guillermo N., Sergio S. P. (2017). Comparison of parasitological techniques for the diagnosis of intestinal parasitic infections in patients with presumptive malabsorption. *Journal of Parasitic Diseases*.

[25] Sow D., Parola P., Sylla K. (2017). Performance of real-time polymerase chain reaction assays for the detection of 20 gastrointestinal parasites in clinical samples from Senegal. *The American Journal of Tropical Medicine and Hygiene*.

[26] Dacal E., Saugar J. M., Soler T. (2018). Parasitological versus molecular diagnosis of strongyloidiasis in serial stool samples: how many?. *Journal of Helminthology*.

[27] Formenti F., La Marca G., Perandin F. (2019). A diagnostic study comparing conventional and real-time PCR for _Strongyloides stercoralis_ on urine and on faecal samples. *Acta Tropica*.

[28] Al-Harthi S. A., Jamjoom M. B. (2007). Enteroparasitic occurrence in stools from residents in southwestern region of Saudi Arabia before and during Umrah season. *Saudi Medical Journal*.

