# Corrigendum to “scFv against HSP60 of *Strongyloides sp.* and Its Application in the Evaluation of Parasite Frequency in the Elderly”

**DOI:** 10.1155/2021/9761898

**Published:** 2021-06-08

**Authors:** Camila Botelho Miguel, Marcelo Arantes Levenhagen, Fabiana de Almeida Araújo Santos, Julia Maria Costa-Cruz, Luiz Ricardo Goulart, Patrícia Terra Alves, Carlos Ueira-Vieira, Patrícia Kellen Martins Oliveira Brito, Angelica Oliveira Gomes, Javier Emilio Lazo-Chica, Carlo José Freire Oliveira, Wellington Francisco Rodrigues

**Affiliations:** ^1^Federal University of Triângulo Mineiro (UFTM), 38061-500 Uberaba, MG, Brazil; ^2^University Center of Mineiros-Unifimes, 75, 830-000 Mineiros, GO, Brazil; ^3^Federal University of Uberlândia, 38400-902, Uberlandia, MG, Brazil; ^4^University of São Paulo, 14049900, Ribeirao Preto, SP, Brazil

In the article titled “scFv against HSP60 of *Strongyloides* sp. and Its Application in the Evaluation of Parasite Frequency in the Elderly” [1], Dr. Fabiana de Almeida Araújo Santos was missing from the author list, who contributed to the data analysis, revision of the manuscript, and approval of the final version for publication. The corrected author list is shown above.
